# Long-term outcomes with HLX01 (HanliKang^®^), a rituximab biosimilar, in previously untreated patients with diffuse large B-cell lymphoma: 5-year follow-up results of the phase 3 HLX01-NHL03 study

**DOI:** 10.1186/s12885-024-11876-9

**Published:** 2024-01-24

**Authors:** Yan Qin, Yongping Song, Dong Wang, Ou Bai, Jifeng Feng, Xiuhua Sun, Lihua Qiu, Jianmin Yang, Yu Yang, Zhao Wang, Jianda Hu, Huaqing Wang, Hang Su, Zhengming Jin, Wenbin Qian, Chuan Jin, Mingzhi Zhang, Ding Yu, Li Liu, Guoan Chen, Yarong Li, Tao Sun, Jie Jin, Huizheng Bao, Xin Du, Hui Zhou, Gan Fu, Yuankai Shi

**Affiliations:** 1 Department of Medical Oncology, Beijing Key Laboratory of Clinical Study on Anticancer Molecular Targeted Drugs,National Cancer Center/National Clinical Research Center for Cancer/Cancer Hospital, Chinese Academy of Medical Sciences & Peking Union Medical College, No. 17 Panjiayuan Nanli, Chaoyang District, 100021 Beijing, China; 2 Department of Hematology, the First Affiliated Hospital of Zhengzhou University, Zhengzhou, China; 3 Department of Oncology, Army Characteristic Medical Center, Chongqing, China; 4 Department of Hematology, Cancer Center, the First Hospital of Jilin University, Changchun, China; 5 Department of Oncology, Jiangsu Cancer Hospital, the Affiliated Cancer Hospital of Nanjing Medical University, Jiangsu Institute of Cancer Research, Nanjing, China; 6 Department of Medical Oncology, the Second Affiliated Hospital of Dalian Medical University, Dalian, China; 7 Department of Lymphoma, Tianjin Medical University Cancer Hospital, Tianjin, China; 8 Department of Hematology, Changhai Hospital, Naval Medical University, Shanghai, China; 9 Department of Lymphoma & Head and Neck Tumors, Fujian Medical University Cancer Hospital, Fuzhou, China; 10 Department of Hematology, Beijing Friendship Hospital, Capital Medical University, Beijing, China; 11 Department of Hematology, Fujian Medical University Union Hospital, Fuzhou, China; 12 Department of Medical Oncology, Tianjin Union Medical Centre of Nankai University, Tianin, China; 13 Department of Lymphoma, the Fifth Medical Center, Chinese PLA General Hospital, Beijing, China; 14 Department of Hematology, the First Affiliated Hospital of Soochow University, Suzhou, China; 15 Department of Hematology, the Second Affiliated Hospital, School of Medicine, Zhejiang University, Hangzhou, China; 16 Department of Oncology, Guangzhou Medical University, Guangzhou, China; 17 Department of Oncology, the First Affiliated Hospital of Zhengzhou University, Zhengzhou, China; 18 Department of Oncology Medicine, Hubei Cancer Hospital, Wuhan, China; 19 Department of Hematology, the Second Affiliated Hospital of Air Force Medical University (Tangdu Hospital), Xian, China; 20 Department of Hematology, the First Affiliated Hospital of Nanchang University, Nanchang, China; 21 Hematology and Oncology Department, the Second Hospital of Jilin University, Changchun, China; 22 Department of Breast Medicine, Liaoning Cancer Hospital, Shenyang, China; 23 Department of Hematology, the First Affiliated Hospital of Medical College of Zhejiang University, Hangzhou, China; 24 Department of Lymphology and Hematology, Jilin Provincial Cancer Hospital, Changchun, China; 25 Department of Hematology, Guangdong Provincial People’s Hospital, Guangzhou, China; 26 Department of Lymphoma & Hematology, Hunan Cancer Hospital, Changsha, China; 27 Department of Hematology, Xiangya Hospital, Central South University, Changsha, China

**Keywords:** HLX01, Rituximab biosimilar, DLBCL, Overall survival, HanliKang^®^

## Abstract

HLX01 (HanliKang^®^) is a rituximab biosimilar that showed bioequivalence to reference rituximab in untreated CD20-positive diffuse large B-cell lymphoma (DLBCL) in the phase 3 HLX01-NHL03 study. Here, we report the 5-year follow-up results from the open-label extension part. Patients were randomised to either rituximab plus cyclophosphamide, doxorubicin, vincristine, and prednisone (R-CHOP) or HLX01 plus CHOP (H-CHOP) every 21 days for up to six cycles. The primary efficacy endpoint was overall survival (OS), and secondary efficacy endpoint was progression-free survival (PFS). Of the 407 patients enrolled in HLX01-NHL03, 316 patients (H-CHOP = 157; R-CHOP = 159) were included in the 5-year follow-up for a median duration of 65.1 (range, 2.2–76.5) months. 96.5% of the patients had an International Prognostic Index (IPI) of 1 or 2, and 17.7% had bone marrow involvement. The 5-year OS rates were 81.0% (95% CI: 74.9–87.5%) and 75.4% (95% CI: 68.9–82.6%)( HR: 0.75, 95% CI 0.47–1.20; *p* = 0.23) while 5-year PFS rates were 77.7% (95% CI: 71.4–84.6%) and 73.0% (95% CI: 66.3–80.3%) (HR: 0.84, 95% CI 0.54–1.30; *p* = 0.43) in the H-CHOP and R-CHOP groups, respectively. Treatment outcomes did not differ between groups regardless of IPI score and were consistent with the primary analysis. H-CHOP and R-CHOP provided no significant difference in 5-year OS or PFS in previously untreated patients with low or low-intermediate risk DLBCL.

**Supplementary Information:**

The online version contains supplementary material available at 10.1186/s12885-024-11876-9.

## Introduction

Diffuse large B-cell lymphoma (DLBCL) is the most common type of lymphoid malignancy, accounting for 25–30% of all non-Hodgkin’s lymphoma [1]. In China, DLBCL accounts for more than one-third of lymphoid neoplasms [2]. Rituximab is a chimeric anti-CD20 monoclonal antibody directed against the CD20 antigen on the surface of B lymphocytes [3]. Rituximab destroys malignant B lymphocytes by inducing complement-dependent cytotoxicity and antibody-dependent cell-mediated cytotoxicity or phagocytosis and apoptosis [3]. 

Although DLBCL is an aggressive tumour, patients respond well with rituximab plus cyclophosphamide, doxorubicin, vincristine, and prednisone (R-CHOP) [1], achieving a higher complete response than with CHOP alone (76% vs. 63%; *p* = 0.005) in the pivotal LNH-98.5 trial [4]. The long-term follow-up of the same study showed that the median OS in the R-CHOP arm (8.4 years [95% CI: 5.4–not reached]) was significantly prolonged than that in the CHOP arm (3.5 years [95% CI: 2.2–5.5]) (*p* < 0.0001), further confirming the long-term benefit of R-CHOP regimen [5]. Nowadays, rituximab in combination with chemotherapy remains the mainstay of treatment for this lymphoma subtype, representing a standard of care in the first-line setting with a curative intent.

The clinical benefit of the R-CHOP regimen is also well documented in Chinese patients. First-line treatment with R-CHOP in Chinese patients with DLBCL yielded an objective response rate of 94.2% in a real-world study [6]. In a retrospective study of 411 patients, the overall response rate in R-CHOP was higher than that in CHOP alone (95.2% vs. 88.0%; *p* = 0.007) [7]. The Clinical practice guideline for lymphoma in China (2021 Edition) recommends anti-CD20 antibody plus chemotherapy for newly diagnosed patients with DLBCL [8]. 

Biosimilars are biological agents that are highly similar to the active ingredient of the reference biologic in terms of structure, pharmacokinetics, pharmacodynamics, efficacy, and safety. The process of developing a biosimilar includes structural and functional characterisation of the molecule, preclinical studies, and clinical studies, with the goal of proving no clinically meaningful difference between the biosimilar and the reference product [9]. To make rituximab more readily available, biosimilars are developed. HLX01 (HanliKang^®^; Shanghai Henlius Biotech, Inc., China) is a rituximab biosimilar that has demonstrated bioequivalence in terms of physicochemical properties and biological activity to the reference rituximab [10]. It was developed in a stepwise approach in accordance with the China National Medical Products Administration (NMPA) and the World Health Organization similar biotherapeutic product development guidelines [11]. 

In a phase 1 study, HLX01 and reference rituximab showed bioequivalence in terms of pharmacokinetics and pharmacodynamics in patients with CD20-positive B-cell lymphoma [11]. The efficacy and safety of HLX01, and bioequivalence to the reference rituximab in DLBCL, has also been demonstrated in a phase 3 HLX01-NHL03 trial of 407 treatment-naïve patients with low to intermediate risk (International Prognostic Index [IPI] 0–2) [12]. In order to have a consistent treatment plan in a clinical trial setting (i.e., H-CHOP or R-CHOP every 21 days for up to six cycles), patients with higher IPI scores of ≥ 3 were not recruited in this study as they would require R-CHOP at higher intensity or for extended cycles (e.g., eight cycles) [8]. The overall response rates for HLX01 plus CHOP (H-CHOP) group and R-CHOP group were 94.1% (95% CI: 89.8–97.0%) and 92.8% (95% CI: 88.2–96.0%), respectively (intergroup difference, 1.4%; 95% CI,−3.59 to 6.32, *p* = 0.608) in the per protocol set [12]. More recently, HLX01 demonstrated comparable efficacy compared with the reference rituximab in a real-world study, yielding an overall response rate of 86.7% versus 88.9% for the reference product in Chinese patients (*p* = 1.000) [13]. HLX01 has been approved by China NMPA as the first biosimilar in China on 22 February 2019 [14–16]. 

Here, we report the results from the 5-year follow-up analyses on the OS of the HLX01-NHL03 phase 3 study [12]. 

## Methods

### Study design and patient eligibility

In the phase 3, multicentre, randomised, double-blind HLX01-NHL03 study, patients were randomised to receive either H-CHOP or R-CHOP at a dose of 375 mg/m^2^ for HLX01 or rituximab intravenously once every three weeks on a three-week cycle for up to six cycles. In the open-label extension part, the enrolled patients were those randomised in the HLX01-NHL03 study and were willing to be followed up for survival, disease progression, and treatment status. As such, the eligibility criteria are that of the HLX01-NHL03 study (chinadrugtrials.org.cn, identifier CTR20150583), which has been published previously [12]. 

Key inclusion criteria included treatment-naïve CD20-positive DLBCL patients confirmed by histopathology, IPI of 0–2, and with an expected survival of more than 6 months. Patients were excluded if they had central nervous system (CNS) lymphoma and secondary CNS invasion, double or triple hit DLBCL, or a history of other malignant tumours other than skin squamous cell carcinoma, skin basal cell carcinoma, and cervical carcinoma in situ. The full eligibility criteria are available in the Supplementary Methods.

All patients who agreed to participate in this extended phase were contacted every 3 months (± 7 days) or per routine clinical follow-up until the patient or the legal guardian voluntarily requested to withdraw or was deemed unsuitable to continue in the study by the investigator. This study was conducted in accordance with the International Conference on Harmonization Good Practice for Clinical Trials and local applicable regulatory requirements. The study protocol, amendments, and all related materials were approved by the independent review board at each participating hospital. The study was registered with ClinicalTrials.gov, NCT04491721.

### Study endpoints

The primary efficacy endpoint of this extended follow-up study was to evaluate the 5-year OS from the HLX01-NHL03 study. The secondary efficacy endpoint was PFS.

### Statistical analysis

The OS, PFS, their median values and 95% CI, were calculated using the Kaplan–Meier method. Comparison between groups was performed using the log-rank test. The 1-year, 3-year, and 5-year survival rates were also estimated using the Kaplan–Meier method; comparison between groups was performed using Chi-square test. Efficacy was analysed in patients who participated in the extended follow-up and in those who had completed six cycles of treatment. All statistical analyses were performed using the SAS statistical software, version 9.4 or above (SAS Institute, Inc., Cary, NC). All hypothesis tests were two-sided, using a test level of 0.05, and the reliability of all CIs was 95%.

## Results

### Patients

**Fig. 1:**
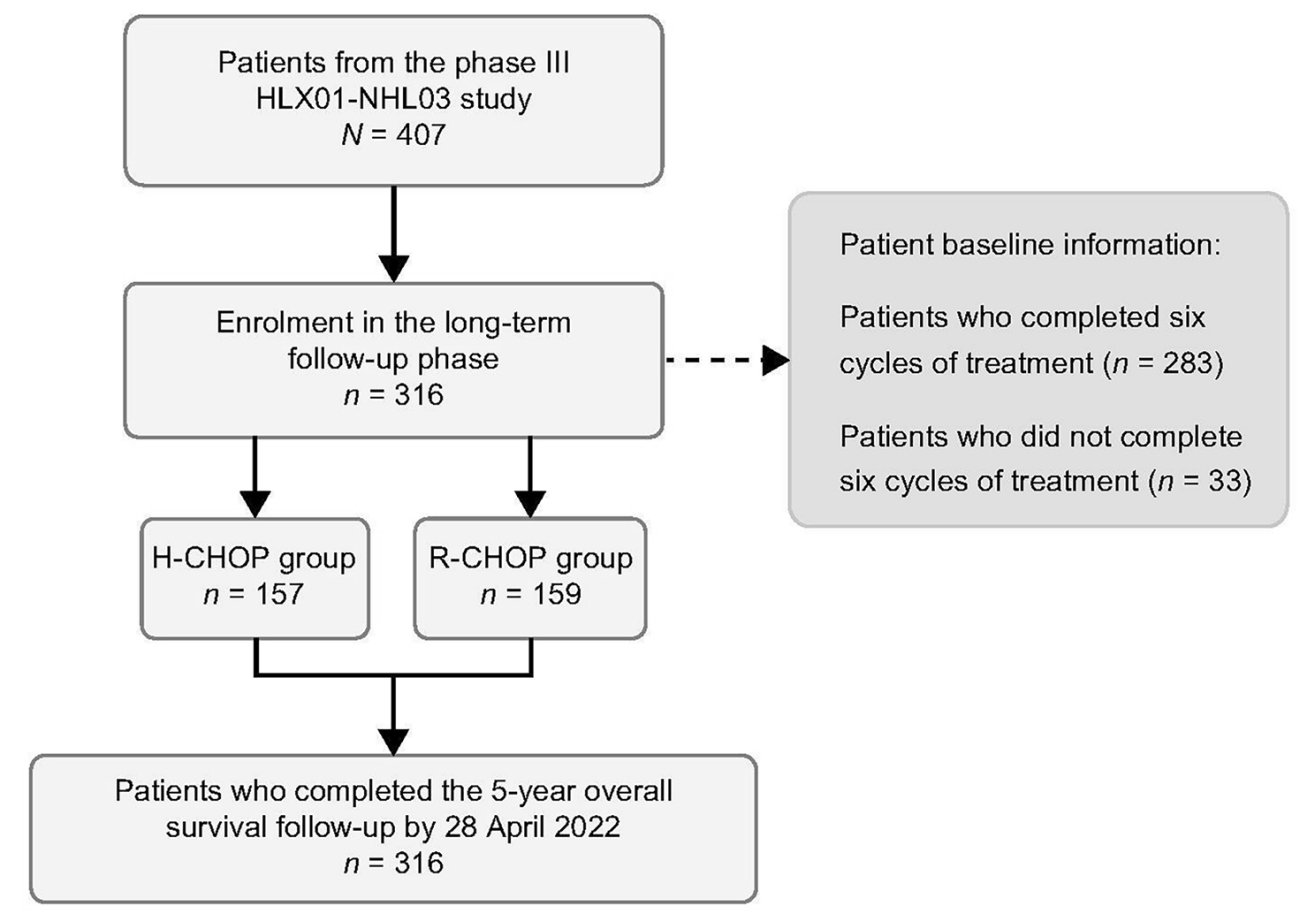
Patient disposition in the long-term follow-up phase

A total of 407 patients were included in the HLX01-NHL03 study; of whom 316 patients (H-CHOP, *n* = 157; R-CHOP, *n* = 159) from 27 hospitals in China were enrolled in this 5-year follow-up phase. At data cut-off date on 28 April 2022, the median duration of follow-up was 65.1 (range, 2.2–76.5) months. Of patients enrolled, 137 (87.3%) in the H-CHOP group and 146 (91.8%) in the R-CHOP group completed the six planned treatment cycles (Fig. 1). The patient demographics and baseline characteristics were well balanced between both treatment groups and are presented in Table 1. The median age of patients from this extended follow-up study was 56.1 years old; 305 patients (96.5%) were IPI 1 and 2, 149 (47.2%) were clinical stage III/IV, and 56 (17.7%) had bone marrow involvement.

**Table 1:** Patient baseline demographics and disease characteristics

	HLX01-NHL03 study			5-year follow-up study		
Characteristic	H-CHOP (*N* = 199)	R-CHOP (*N* = 203)	Total (*N* = 402)	H-CHOP (*N* = 157)	R-CHOP (*N* = 159)	Total (*N* = 316)
Median age (range), year	54 (46–61)	55 (46–63)	NR	56.9 (23.9–76.2)	55.5 (24.4–76.6)	56.1 (23.9–76.6)
Sex						
Male	118 (59.3)	102 (50.2)	220 (54.7)	93 (59.2)	79 (49.7)	172 (54.4)
Female	81 (40.7)	101 (49.8)	182 (45.3)	64 (40.8)	80 (50.3)	144 (45.6)
ECOG PS						
0	75 (37.7)	75 (36.9)	150 (37.3)	63 (40.1)	65 (40.9)	128 (40.5)
1	94 (47.2)	96 (47.3)	190 (47.3)	70 (44.6)	70 (44.0)	140 (44.3)
2	30 (15.1)	32 (15.8)	62 (15.4)	24 (15.3)	24 (15.1)	48 (15.2)
IPI						
0	8 (4.0)	7 (3.4)	15 (3.7)	5 (3.2)	5 (3.1)	10 (3.2)
1	95 (47.7)	106 (52.2)	201 (50.0)	70 (44.6)	83 (52.2)	153 (48.4)
2	96 (48.2)	90 (44.3)	186 (46.3)	82 (52.2)	70 (44.0)	152 (48.1)
3	0 (0.0)	0 (0.0)	0 (0.0)	0 (0.0)	1 (0.6)	1 (0.3)
Clinical stage						
I	20 (10.1)	28 (13.8)	48 (11.9)	16 (10.2)	27 (17.0)	43 (13.6)
II	82 (41.2)	84 (41.4)	166 (41.3)	63 (40.1)	61 (38.4)	124 (39.2)
III	62 (31.2)	62 (30.5)	124 (30.8)	48 (30.6)	52 (32.7)	100 (31.6)
IV	35 (17.6)	29 (14.3)	64 (15.9)	30 (19.1)	19 (11.9)	49 (15.5)
Bone marrow involvement						
Yes	34 (17.1)	32 (15.8)	66 (16.4)	30 (19.1)	26 (16.4)	56 (17.7)
No	165 (82.9)	171 (84.2)	336 (83.6)	127 (80.9)	133 (83.6)	260 (82.3)

Note: Data are presented in median (interquartile range) or *n* (%), unless otherwise stated. Percentages may not add up to 100% because of rounding.

Abbreviations: ECOG PS, Eastern Cooperative Oncology Group performance status; IPI, International Prognostic Index; NR, not reported.

### Efficacy in the overall population

**Fig. 2:**
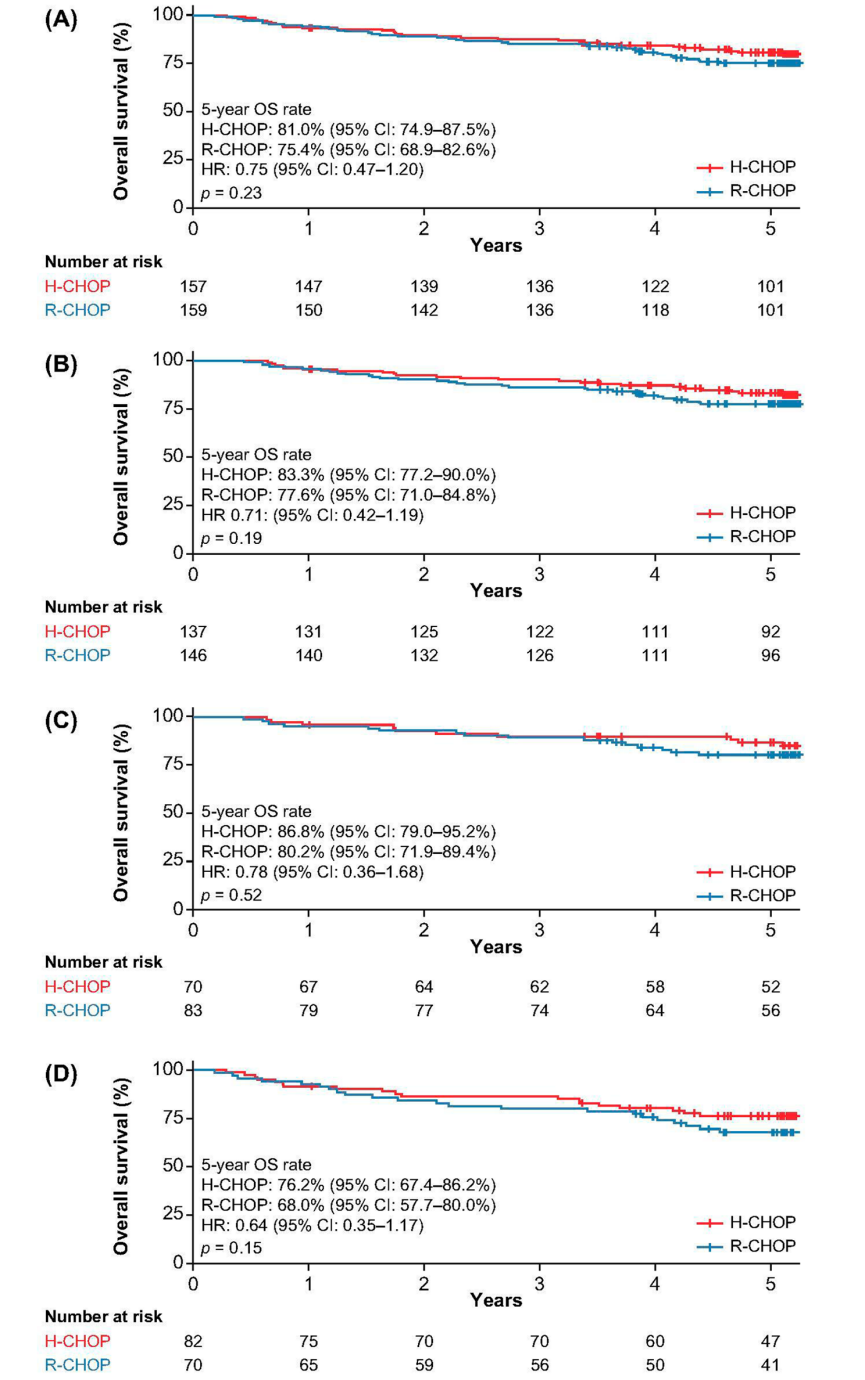
Kaplan–Meier estimates of overall survival in (**A**) the overall population, (**B**) patients who completed six cycles of treatment, (**C**) patients with an IPI score of 1, and (**D**) patients with an IPI score of 2

Thirty-one and 41 patients died in the H-CHOP and R-CHOP groups, respectively. Among 316 patients, there was no statistically significant difference in terms of OS between the two treatment groups. The estimated 5-year OS rates were 81.0% (95% CI: 74.9–87.5%) and 75.4% (95% CI: 68.9–82.6%) in H-CHOP and R-CHOP groups, respectively (HR: 0.75, 95% CI 0.47–1.20; *p* = 0.23; Fig. 2A). The detailed 1-, 3-, and 5-year OS rates are shown in Table 2. OS analysis in 283 patients who completed six treatment cycles at baseline is presented in Fig. 2B. Similarly, no significant difference was observed between both treatment groups. The 5-year OS rates were 83.3% (95% CI: 77.2–90.0%) and 77.6% (95% CI: 71.0–84.8%) for those who completed six treatment cycles in H-CHOP and R-CHOP groups, respectively (HR: 0.71, 95% CI 0.42–1.19; *p* = 0.19).

**Table 2:** 1-, 3-, and 5-year survival rate of H-CHOP and R-CHOP for the overall population, those who had completed 6 cycles of treatment, IPI 1, and IPI 2

	Survival rate (95% CI)		OS		PFS		
			H-CHOP	R-CHOP	H-CHOP		R-CHOP
**Overall** **population**	1-year		93.6 (89.9–97.5)	94.3 (90.8–98.0)	92.3 (88.3–96.6)		91.8 (87.7–96.2)
	3-year		87.8 (82.8–93.1)	85.5 (80.2–91.2)	83.2 (77.6–89.3)		82.4 (76.7–88.5)
	5-year		81.0 (74.9–87.5)	75.4 (68.9–82.6)	77.7 (71.4–84.6)		73.0 (66.3–80.3)
**Patients who had completed 6 cycles of treatment**	1-year		95.6 (92.3–99.1)	95.9 (92.7–99.2)	94.1 (90.3–98.2)		93.8 (90.0-97.8)
	3-year		90.4 (85.6–95.5)	86.3 (80.9–92.1)	85.2 (79.4–91.4)		83.6 (77.8–89.8)
	5-year		83.3 (77.2–90.0)	77.6 (71.0-84.8)	79.6 (73.0-86.8)		75.6 (68.9–83.0)
**IPI 1**	1-year		95.7 (91.1–100)	95.2 (90.7–99.9)	94.3 (89.0-99.9)		95.2 (90.7–99.9)
	3-year		89.9 (83.1–97.3)	89.2 (82.7–96.1)	84.1 (76.0-93.2)		85.5 (78.3–93.5)
	5-year		86.8 (79.0-95.2)	80.2 (71.9–89.4)	84.1 (76.0-93.2)		75.3 (66.4–85.3)
**IPI 2**	1-year		91.5 (95.6–97.7)	92.9 (87.0-99.1)	90.2 (84.0-96.9)		87.1 (79.6–95.3)
	3-year		86.5 (79.4–94.3)	80.0 (71.2–89.9)	82.7 (74.8–91.4)		87.1 (67.9–87.6)
	5-year		76.2 (67.4–86.2)	68.0 (57.7–80.0)	72.0 (62.6–82.7)		68.2 (58.0-80.1)

**Fig. 3:**
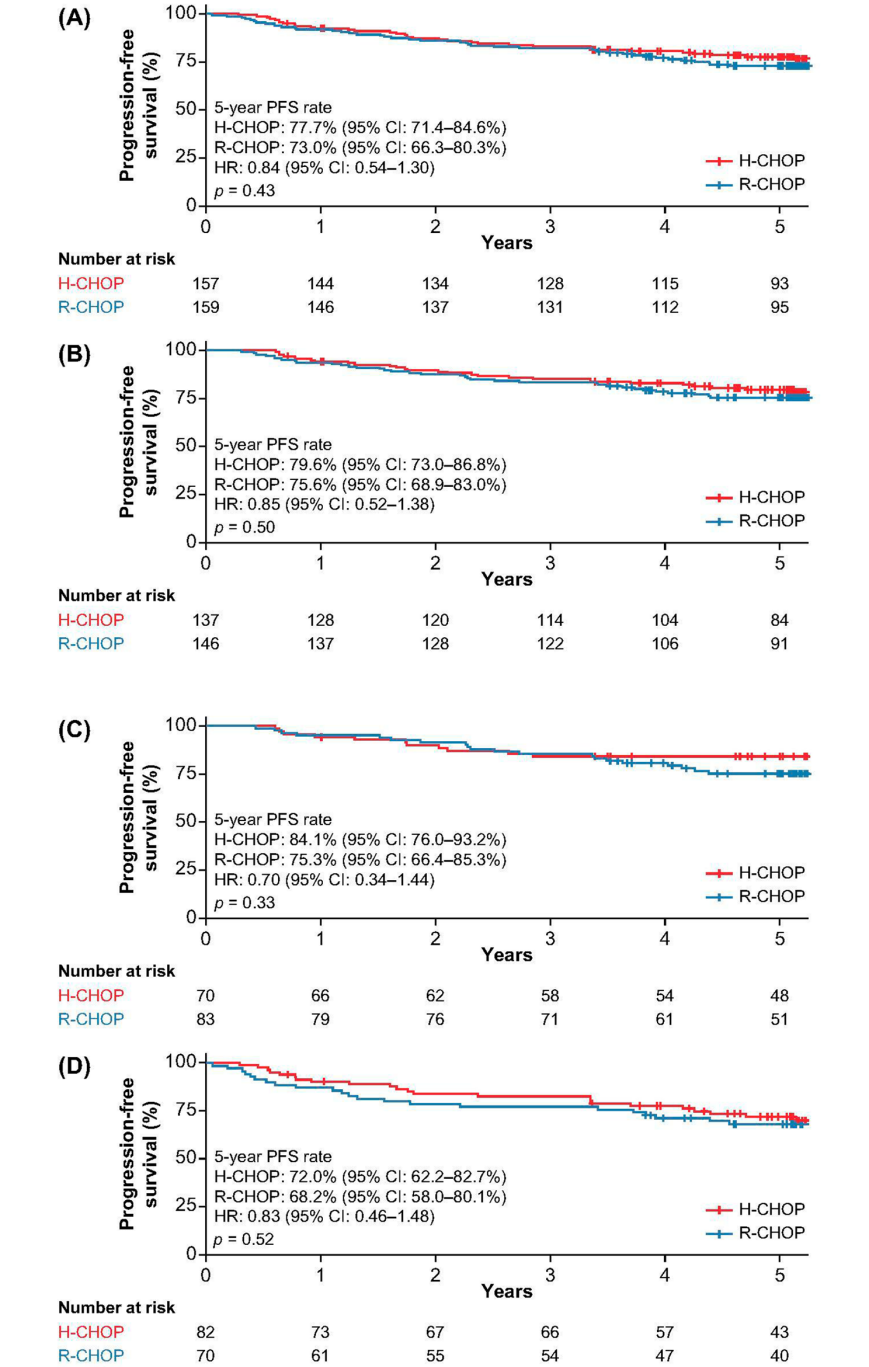
Kaplan–Meier estimates of progression-free survival in (**A**) the overall population, (**B**) patients who completed six cycles of treatment, (**C**) patients with an IPI score of 1, and (**D**) patients with an IPI score of 2

Thirty-six and 43 patients had disease progression or died in the H-CHOP and R-CHOP groups, respectively. There was no statistically significant difference in terms of PFS between the two treatment regimens. The estimated 5-year PFS rates were 77.7% (95% CI: 71.4–84.6%) and 73.0% (95% CI: 66.3–80.3%) in the H-CHOP and R-CHOP groups, respectively (HR: 0.84, 95% CI 0.54–1.30; *p* = 0.43; Fig. 3A). The detailed 1-, 3-, and 5-year PFS rates were shown in Table 2. PFS analyses in 283 patients who completed six cycles of treatment at baseline showed no significant difference in the H-CHOP group versus the R-CHOP group. The 5-year PFS rates were 79.6% (95% CI: 73.0–86.8%) and 75.6% (95% CI: 68.9–83.0%) for those who completed six treatment cycles in H-CHOP and R-CHOP groups, respectively (HR: 0.85, 95% CI 0.52–1.38; *p* = 0.50, Fig. 3B). The detailed 1-, 3-, and 5-year PFS rates are shown in Table 2.

### Subgroup analyses of efficacy outcomes according to IPI and gender

Further analysis was conducted among patients with an IPI score of 1 and 2 as this group of patients made up the majority (*n* = 305; 96.6%) and representing most of the low– and low-intermediate–risk groups in this study. There was no significant difference between H-CHOP and R-CHOP in terms of OS regardless of whether the patients had an IPI score of 1 (5-year OS rate: 86.8% [95% CI: 79.0–95.2%] vs. 80.2% [95% CI: 71.9–89.4%]; HR: 0.78 [95% CI:0.36–1.68]; *p* = 0.52; Fig. 2C) or 2 (5-year OS rate: 76.2% [95% CI: 67.4–86.2%] vs. 68.0% [95% CI: 57.7–80.0%]; HR: 0.64 [95% CI: 0.35–1.17]; *p* = 0.15; Fig. 2D). The detailed 1-, 3-, and 5-year OS rates are shown in Table 2. Similarly, PFS did not differ significantly between H-CHOP and R-CHOP whether the patients had an IPI score of 1 (5-year PFS rate: 84.1% [95% CI: 76.0–93.2%] vs. 75.3% [95% CI: 66.4–85.3%]; HR: 0.70 [95% CI: 0.34–1.44]; *p* = 0.33; Fig. 3C) or 2 (5-year PFS rate: 72.0% [95% CI: 62.2–82.7%] vs. 68.2% [95% CI: 58.0–80.1%]; HR: 0.83 [95% CI: 0.46–1.48]; *p* = 0.52; Fig. 3D). The detailed 1-, 3-, and 5-year PFS rates are shown in Table 2.

There was no significant difference in OS between the two treatment groups among patients with an IPI score of 1 and 2 (*p* = 0.17; Figure S1A); 5-year OS rates were 81.0% (95% CI: 74.9–87.6%) and 74.6% (95% CI: 67.9–81.9%) in the H-CHOP and R-CHOP groups, respectively (Supplementary Table S1). Similar result was also observed with PFS between H-CHOP and R-CHOP among patients with IPI score of 1 and 2 (*p* = 0.34; Figure S1B). The 5-year PFS rates were 77.7% (95% CI: 71.2–84.7%) and 72.0% (95% CI: 65.1–79.6%) in the H-CHOP and R-CHOP groups, respectively (Supplementary Table S1).

OS (*p* = 0.023) and PFS (*p* = 0.048) were significantly different between patients with an IPI score of 1 and those with an IPI score of 2 regardless of treatment regimen (Figure S2). Higher OS and PFS rates were observed in patients with IPI score of 1 compared with those with IPI score of 2. The 5-year OS rates were 83.1% (95% CI: 77.3–89.4%) and 72.3% (95% CI: 65.5–79.9%), and 5-year PFS rates were 79.3% (95% CI: 73.1–86.1%) and 70.2% (95% CI: 63.1–78.0%) in the IPI 1 and 2 groups, respectively (Supplementary Table S1).

The impact of clinical staging on survival outcomes among patients with IPI score of 1 and 2 was further investigated. There was no statistical difference between patients with clinical stage I/II and those with clinical stage III/IV in terms of PFS (*p* = 0.06) and OS (*p* = 0.45) (Figure S3). The 5-year PFS rates were 79.0% (95% CI: 72.8–85.8%) and 70.4% (95% CI: 63.3–78.2%); similarly, 5-year OS rates were 79.4% (95% CI: 73.1–86.2%) and 76.0% (95% CI: 69.4–83.3%) in the clinical stage I/II and III/IV, groups respectively.

Gender appeared to have no effect on either OS or PFS, and the efficacy outcomes did not differ significantly between two treatment groups in either gender group (Figure S4 and Figure S5).

## Discussion

To our knowledge, this is the first rituximab biosimilar study to provide long-term efficacy insights in Chinese patients with low to intermediate risk (IPI score 0–2) DLBCL. The results in this study concur with the findings from the primary analysis, that there was no significant difference in terms of efficacy between both treatment groups [12]. Overall, HLX01 was comparable with the rituximab reference product as the survival rates in terms of OS and PFS were similar without statistical difference. After a median follow-up of 65.1 months, we noted a trend towards OS and PFS benefit although no statistical difference, the 5-year OS rate (study primary endpoint) between H-CHOP group and R-CHOP group (81.0% [95% CI: 74.9–87.5%] vs. 75.4% [95% CI: 68.9–82.6%]; HR: 0.75, 95% CI 0.47–1.20; *p* = 0.23); similar results were also observed for the 5-year PFS rate between H-CHOP group and R-CHOP group (77.7% [95% CI: 71.4–84.6%] vs. 73.0% [95% CI: 66.3–80.3%]; HR: 0.84, 95% CI 0.54–1.30; *p* = 0.43). In addition, the results of the OS and PFS subgroup analyses stratified by IPI status, gender, or the completion of six treatment cycles at baseline were consistent with the overall results and yielded no significant difference between treatment groups.

The results in this study were comparable (in view of baseline IPI status and age of patients enrolled) with other long-term follow-up studies with rituximab plus chemotherapy in DLBCL patients. In a retrospective, observational study conducted in China, the OS and PFS of patients who received R-CHOP was 84.1% and 81.5%, respectively, after a median follow-up of 86 months [17]. The majority (75.6%) of these patients had an IPI ≤ 2 and the median age was 53 years [17]. In a multicentre, prospective, non-interventional study in China, 3-year OS and PFS rates with rituximab plus chemotherapy were 90% and 59%, respectively, in previously untreated patients with DLBCL [18]. A large proportion of patients (77.0%) were low– and low-intermediate IPI risk score and median age was 57.2 years [18]. Of note, 92.4% of patients received R-CHOP in this study, while 7.6% were given rituximab monotherapy [18]. Elsewhere, Li et al. reported 3-year OS and PFS of 66.1% and 77.6%, respectively, among Chinese patients with a median age of 54 years who received R-CHOP every 3 weeks [19]. The higher proportion of patients (30.8%) with IPI > 2 could explain the slightly lower survival outcomes reported in this randomised, open-label phase 3 study [19]. When stratified by IPI scores, the 3-year OS and PFS rates were 86.8% and 76.5% for IPI 0–1, and 76.0% and 57.5% for IPI 2, respectively [19]. In a published study on the use of revised-IPI to predict treatment outcomes by Sehn et al., 4-year OS and PFS among patients with 1 or 2 IPI score was 79% and 80%, respectively, similar to the 5-year survival rates in our study where the majority of patients had a IPI score of 1 or 2 [20]. The clinical trial LNH98-5, which was conducted in Europe and included an older population (median age, 69 years) with IPI 0–5, showed a 5-year PFS of 54% and 5-year OS of 58% for R-CHOP [21]. A real-world study in Chile also reported a similar 5-year OS (66%) in an older population (median age 62, range 15–95) with higher risk (IPI 0–5) treated with R-CHOP [22]. The MabThera International Trial (MInT) Group enrolled a younger population (aged 18–60 years) with a favourable prognostic profile (age-adjusted IPI 0–1) and reported a 6-year PFS of 80.2% and 6-year OS of 90.1% for R-CHOP [23]. 

More recently, Shi et al. published the treatment outcomes of R-CHOP in 1084 Chinese patients with DLBCL from the Cancer Hospital, Chinese Academy of Medical Sciences & Peking Union Medical College which reported 5-year OS rates of 86.1% and 59.6% among low–risk and low-intermediate–risk patients by IPI risk categorisation, respectively [24]. The corresponding 5-year PFS rates were 78.3% and 51.8% [24]. Another large-scale retrospective study of 2124 patients reported 5-year OS rates of 87.7% and 76.1% in the IPI 0–1 and 2 risk groups, respectively [25]. Overall, the 5-year OS rates of H-CHOP at 86.8% and 76.2% for patients with IPI score of 1 and 2, respectively in this study were similar to that of previous published studies. IPI score is a prognostic tool to predict the outcome of patients with DLBCL treated with R-CHOP. In the subgroup analysis of this study, patients with IPI 2 exhibited poorer PFS and OS compared with those with IPI 1, regardless of treatment regimen, and this is well documented in literature [19, 20, 24]. 

Interestingly, Chinese patients appeared to have longer survival outcomes than those from western countries when treated with R-CHOP. This could be due to the differences in clinical characteristics such as age of diagnosis which has been documented to be lower in China (50–60 years) than in Caucasian patients (> 60 years) [24, 26–28]. Caucasian patients were also more likely to be presented with elevated serum lactate dehydrogenase and advanced stage than Chinese patients [26, 28], which are prognostic factors for survival outcomes [29]. Further studies are warranted to investigate the differences in prognostic factors between Asian and Caucasian patients and their impact on the long-term survival outcomes of R-CHOP.

The cost-effectiveness of R-CHOP was previously established in China for DLBCL [30]. Given that HLX01 is proven to be effective in the long-term in terms of survival and is relatively inexpensive, the former, therefore, represents a reasonable alternative to the reference rituximab, further improving treatment accessibility, cost-effectiveness, and positively impacting the financial sustainability of the healthcare system.

The current study had several limitations arising from the nature of the study design. Telephone follow-up may be subjected to non-response bias, and the absence of visual cues may compromise the representativeness and robustness of the data. Exploratory subgroup studies are warranted to further understand the impact of prognostic factors such as cell of origin, IPI, or the presence of molecular aberrations on the treatment outcomes of H-CHOP in Chinese patients with DLBCL [31]. 

## Conclusion

This study showed the 5-year OS and PFS rates in previously untreated Chinese DLBCL patients who received H-CHOP was comparable to that of R-CHOP. Rituximab has revolutionised the treatment of DLBCL over the last decades, and HLX01 is an appropriate substitute for rituximab that can provide comparable efficacy in patients with low or low-intermediate IPI risk DLBCL.

### Electronic supplementary material

Below is the link to the electronic supplementary material.

Supplementary Material 1

Supplementary Material 2

Supplementary Material 3

Supplementary Material 4

Supplementary Material 5

Supplementary Material 6

Supplementary Material 7

Supplementary Material 8

Supplementary Material 9

## Data Availability

The data that support the findings of this study are available from the corresponding author upon reasonable request.

## Abbreviations

CI: Confidence interval
CNS: Central nervous system
DLBCL: Diffuse large B-cell lymphoma
H-CHOP: HLX01 plus cyclophosphamide,doxorubicin,vincristine,and prednisone
IPI: International Prognostic Index
OS: overall survival
PFS: Progression-free survival
R-CHOP: Rituximab plus cyclophosphamide,doxorubicin,vincristine,and prednisone
