# Supplementary figures and images for: Long-term outcomes with HLX01 (HanliKang®), a rituximab biosimilar, in previously untreated patients with diffuse large B-cell lymphoma: 5-year follow-up results of the phase 3 HLX01-NHL03 study
Source: BMC Cancer. 2024 Jan 24;24:124. doi: 10.1186/s12885-024-11876-9 (PMC10809427; doi:10.1186/s12885-024-11876-9)

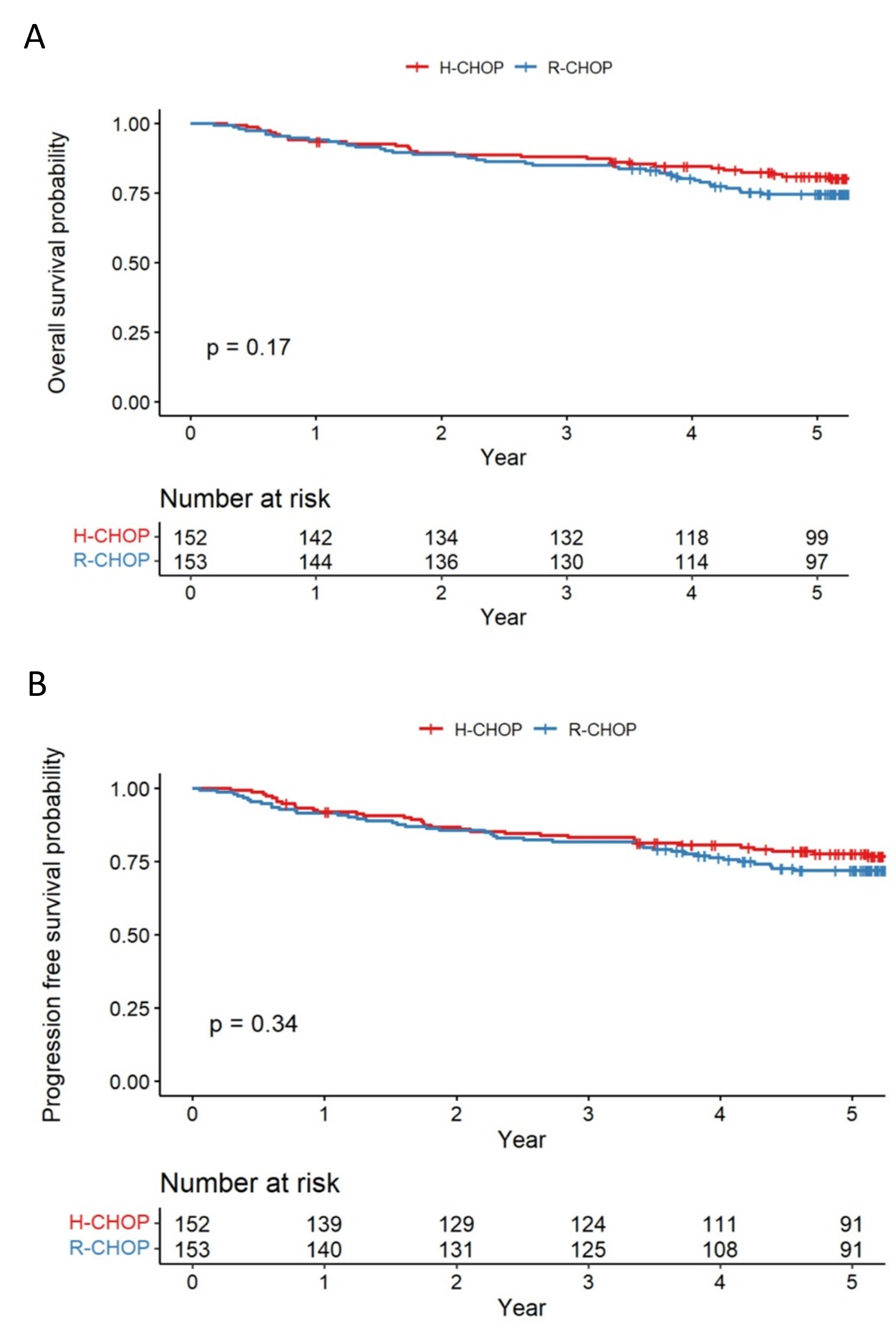

Supplement: Supplementary file 1 — Supplementary Material 1 [file 12885_2024_11876_MOESM1_ESM.tif]

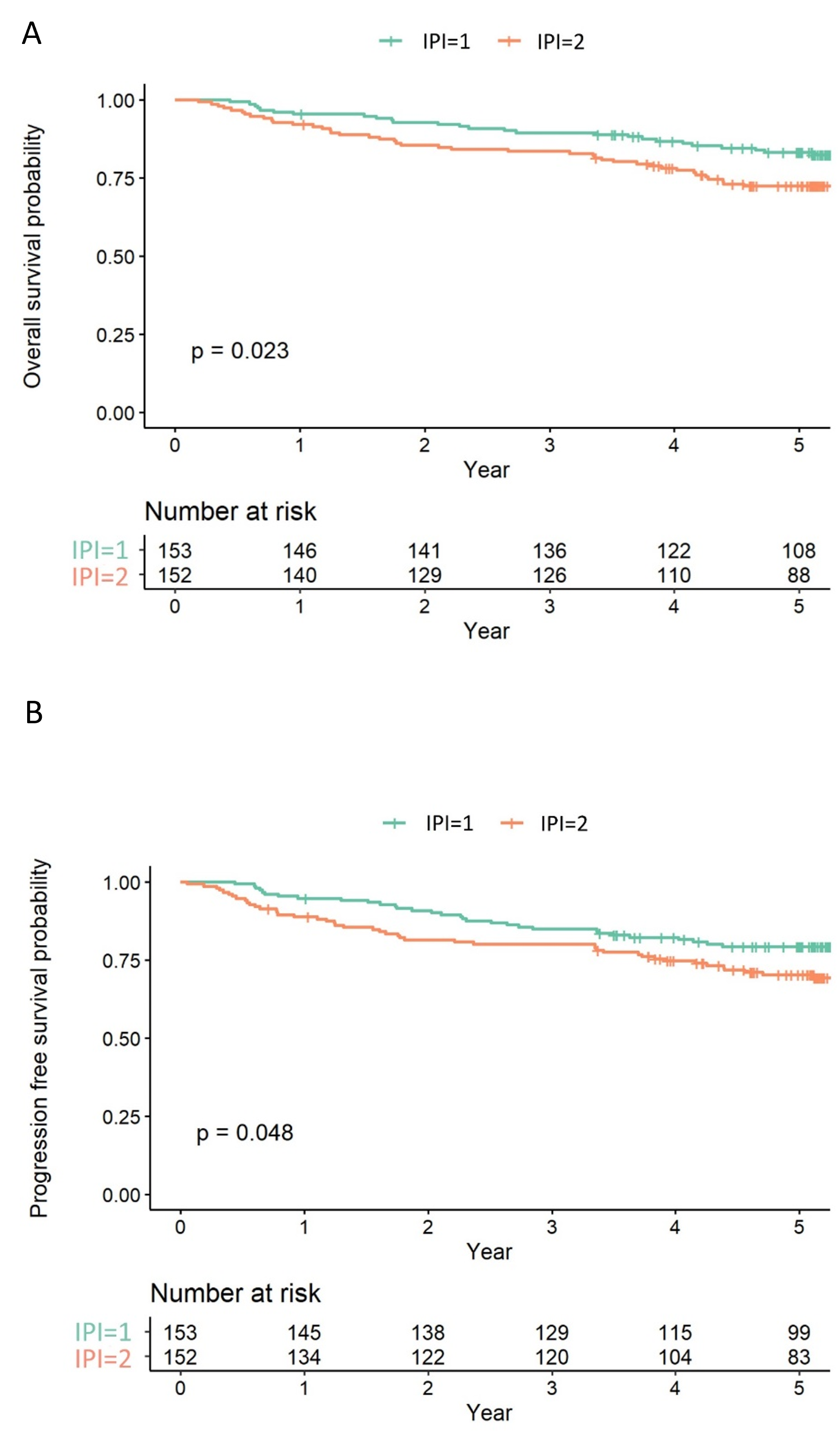

Supplement: Supplementary file 2 — Supplementary Material 2 [file 12885_2024_11876_MOESM2_ESM.tif]

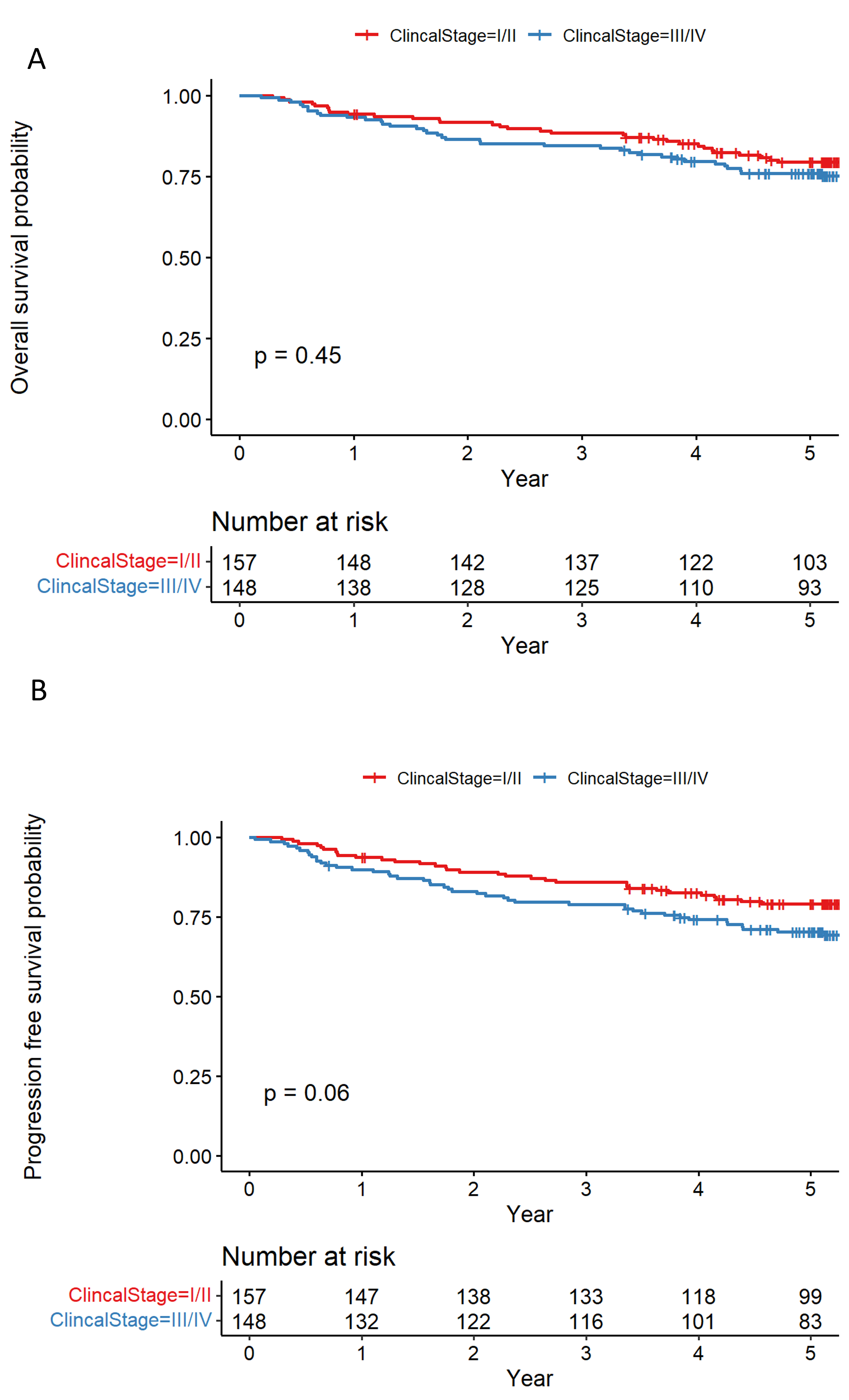

Supplement: Supplementary file 3 — Supplementary Material 3 [file 12885_2024_11876_MOESM3_ESM.tif]

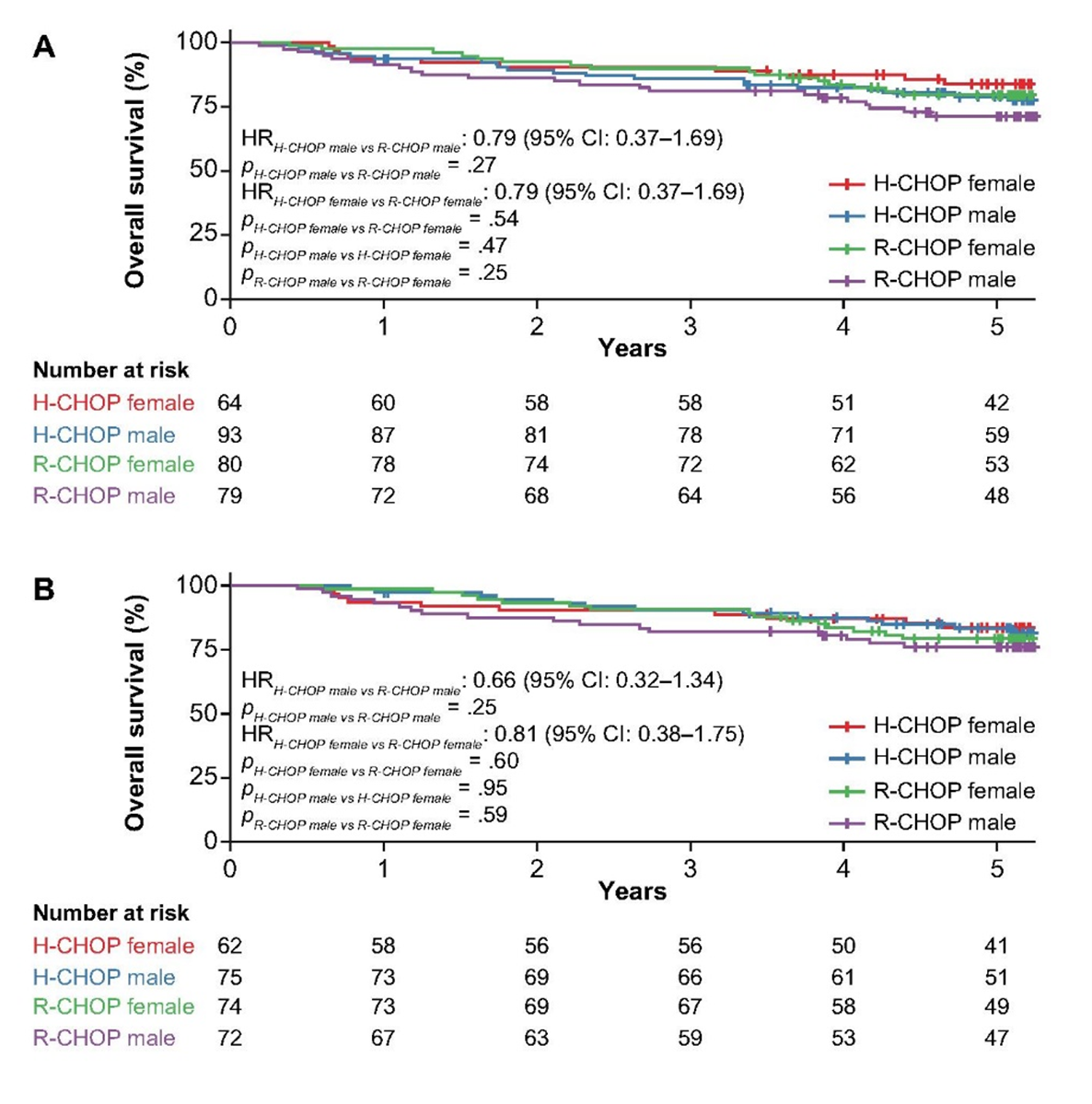

Supplement: Supplementary file 4 — Supplementary Material 4 [file 12885_2024_11876_MOESM4_ESM.tif]

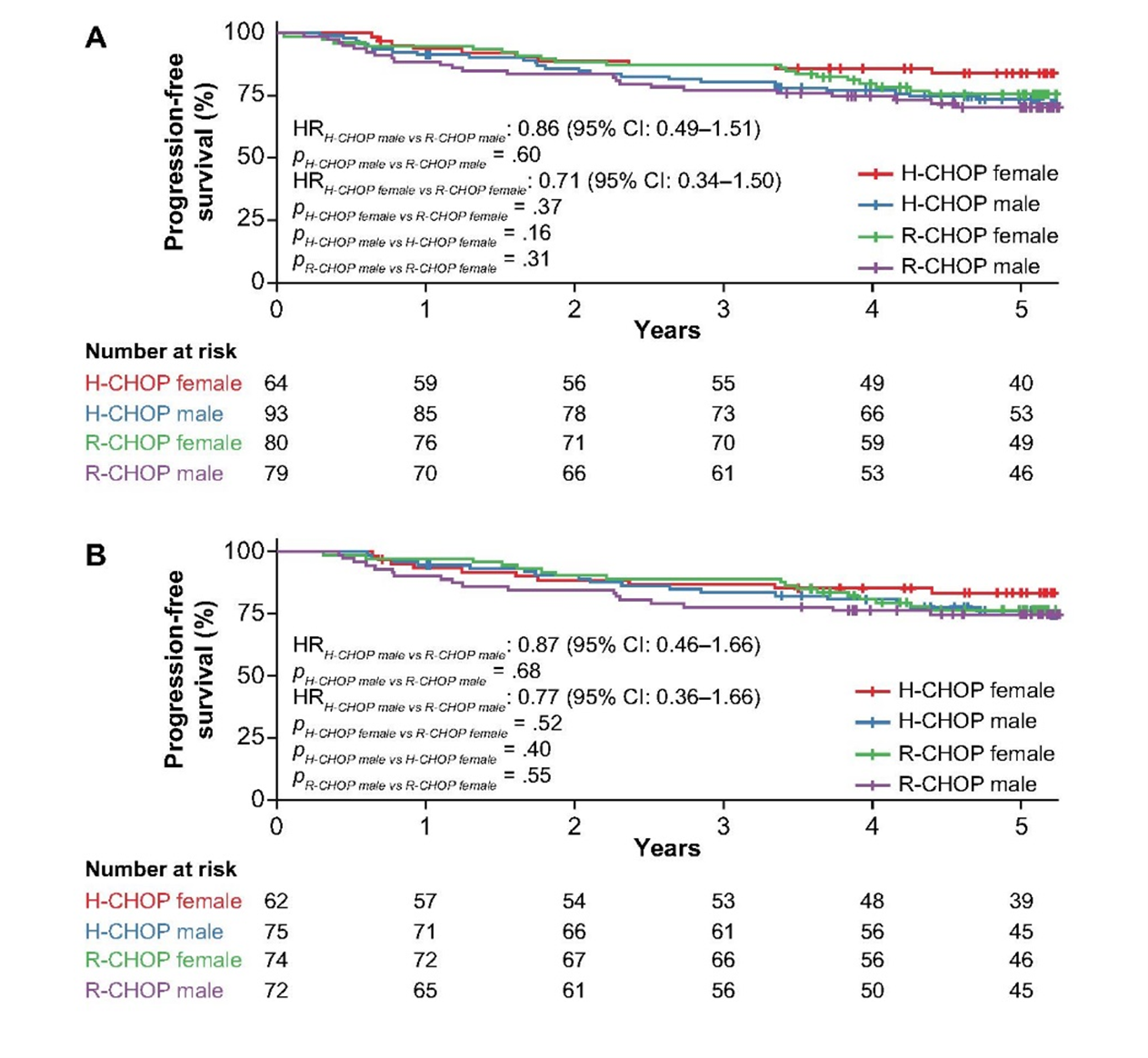

Supplement: Supplementary file 5 — Supplementary Material 5 [file 12885_2024_11876_MOESM5_ESM.tif]
