# Supplementary material for: Long-term outcomes with HLX01 (HanliKang®), a rituximab biosimilar, in previously untreated patients with diffuse large B-cell lymphoma: 5-year follow-up results of the phase 3 HLX01-NHL03 study
Source: BMC Cancer. 2024 Jan 24;24:124. doi: 10.1186/s12885-024-11876-9 (PMC10809427; doi:10.1186/s12885-024-11876-9)
Supplement: Supplementary file 6 — Supplementary Material 6 [file 12885_2024_11876_MOESM6_ESM.docx]

**Legends for supplementary figures**

**Supplementary Figure S1. Kaplan–Meier estimates of (A) overall survival and (B) progression-free survival in patients with IPI score of 1 and 2.**

IPI, International Prognostic Index.

**Supplementary Figure S2.** **Kaplan–Meier estimates of (A) overall survival and (B) progression-free survival in all treated patients stratified by IPI score of 1 or 2.**

IPI, International Prognostic Index.

**Supplementary Figure S3. Kaplan–Meier estimates of (A) overall survival and (B) progression-free survival in all treated patients with IPI score of 1 and 2 stratified by clinical stage.**

IPI, International Prognostic Index.

**Supplementary Figure S4. Kaplan–Meier estimates of overall survival stratified by gender in (A) the overall population and (B) patients who completed six cycles of treatment.**

**Supplementary Figure S5. Kaplan–Meier estimates of progression-free survival when stratified by gender in (A) the overall population and (B) patients who completed six cycles of treatment.**
