# Supplementary material for: Long-term outcomes with HLX01 (HanliKang®), a rituximab biosimilar, in previously untreated patients with diffuse large B-cell lymphoma: 5-year follow-up results of the phase 3 HLX01-NHL03 study
Source: BMC Cancer. 2024 Jan 24;24:124. doi: 10.1186/s12885-024-11876-9 (PMC10809427; doi:10.1186/s12885-024-11876-9)
Supplement: Supplementary file 7 — Supplementary Material 7 [file 12885_2024_11876_MOESM7_ESM.docx]

**Supplementary Table S1. 1-, 3-, and 5-year survival rate of H-CHOP and R-CHOP for patients who had an IPI score of 1 and 2, IPI score of 1 or 2, and IPI score of 1 and 2 stratified by clinical stage**
