# Supplementary material for: Long-term outcomes with HLX01 (HanliKang®), a rituximab biosimilar, in previously untreated patients with diffuse large B-cell lymphoma: 5-year follow-up results of the phase 3 HLX01-NHL03 study
Source: BMC Cancer. 2024 Jan 24;24:124. doi: 10.1186/s12885-024-11876-9 (PMC10809427; doi:10.1186/s12885-024-11876-9)
Supplement: Supplementary file 8 — Supplementary Material 8 [file 12885_2024_11876_MOESM8_ESM.docx]

**Supplementary Method**

Inclusion criteria

1. Newly treated CD20-positive DLBCL confirmed by histopathology and meeting the diagnostic criteria for DLBCL;
2. International Prognostic Index of 0–2 points; patients with stage I–IV disease can be enrolled;
3. Eastern Cooperative Oncology Group performance status 0–2;
4. Expected survival period exceeding 6 months;
5. Age 18–80 years old;
6. At least one two-dimensional measurable lesion as the basis for evaluation: intranodal lesions is defined as long diameter 1.5 cm and short diameter ≥1.0 cm; for extranodal lesions, long diameter should be ≥1.0 cm;
7. Sufficient haematological function during the screening period, that is, WBC ≥4×10^9^/L or ≥ the lower limit of the normal value of the local laboratory, haemoglobin 90g/L, absolute neutrophil count 2×10^9^/L or the normal value of the local laboratory Lower limit, platelet count 100×10^9^/L, patients with bone marrow involvement should have WBC ≥3×10^9^/L, haemoglobin ≥80 g/L, absolute neutrophil count ≥1.5×10^9^/L, platelet count ≥75×10^9^ /L;
8. The results of liver function tests in the screening period meet the following criteria: total bilirubin ≤1.5×upper limits of normal (ULN); ALT or AST ≤2.5×ULN; alkaline phosphatase ≤3×ULN in patients with non-bone invasion; abnormal renal function (serum creatinine ≤ 1.5 times normal);
9. Negative serum HIV antigen or antibody;
10. Negative HCV antibody, or positive HCV antibody, but negative HCV RNA;
11. HBV surface antigen and HBV core antibody are both negative, if any of the above is positive, the peripheral blood hepatitis B virus DNA titer test is required, and the number of copies less than 1×10^3^ copies/mL can be included in the group.

Exclusion criteria

1. Primary central nervous system (CNS) lymphoma and secondary CNS invasion, gray zone lymphoma between Burkitt and DLBCL, grey zone lymphoma between DLBCL and Hodgkin's lymphoma, primary mediastinal DLBCL, Patients with primary exudative lymphoma, plasmablastic lymphoma, primary cutaneous DLBCL, *ALK*-positive DLBCL, transformed lymphoma;
2. Double (*BCL 2* and *c-MYC* gene rearrangement) or triple (*BCL-2*, *BCL-6* and *c-MYC* gene rearrangement) hit diffuse large B-cell lymphoma confirmed by FISH assay. Pathological immunohistochemical test results were: *BCL-2* ≥70% positive and *c-MYC* ≥40% positive, and the tumour cells were judged to be of germinal centre origin according to Han's evaluation criteria, but no clear FISH test results could be obtained;
3. In the past 5 years, there is a history of other malignant tumours other than skin squamous cell carcinoma, skin basal cell carcinoma, and cervical carcinoma in situ;
4. Major surgery (excluding diagnostic surgery) within the past 2 months;
5. Previous NHL treatment:
   - Including chemotherapy or immunotherapy;
   - Radiation therapy (except local radiation therapy);
   - Monoclonal antibody therapy (including rituximab, HLX01, GA101, or any other rituximab biosimilar;
   - surgical treatment (except biopsy);
6. Previously received cytotoxic drugs or anti-CD20 antibodies to treat other diseases (such as rheumatoid arthritis);
7. Any monoclonal antibody has been used within 3 months before enrolment;
8. Those who have participated in other clinical trials and have used other trial-related drugs within 3 months before enrolment;
9. Those who have been vaccinated with (attenuated) live virus vaccine within 1 month before enrolment;
10. Hematopoietic cytokines, such as granulocyte colony stimulating factor, have been used within 2 weeks before enrolment;
11. To control lymphoma symptoms, use a maximum dose of 100 mg of prednisone or equivalent for more than 5 days; use prednisone 30 mg or corticosteroid equivalents daily; for patients receiving prednisone ≤30 mg daily or its corticosteroid equivalent, there must be documented evidence of dose stabilisation for at least 4 weeks prior to randomisation (Day 1 of Cycle 1);
12. Have peripheral nervous system or central nervous system diseases;
13. Suspected active or latent tuberculosis patients;
14. Known active bacterial, viral, fungal, mycobacterial, parasitic, or other infection (excluding nail bed fungal infection) or any major systemic infection requiring intravenous antibiotic treatment or hospitalisation within 4 weeks prior to enrolment event. Related to the completion of the course of antibiotic therapy, except for the treatment of neoplastic fever;
15. Diseases that, in the opinion of the investigator, may limit participation in this trial (eg, severe cardiac insufficiency; myocardial obstruction or malignant arrhythmia or unstable angina pectoris within the last 6 months; gastric ulcer; active autoimmune disease; severe hypertension as determined by the investigator, etc.);
16. Contraindications to any drugs contained in the CHOP regimen, including previous treatment with anthracyclines;
17. Known allergy to the active ingredients, excipients, mouse-derived products, or xenogeneic proteins of any drugs included in this study (including those in the CHOP regimen);
18. Those who are considered by the investigators to be unsuitable for enrolment due to alcohol or drug abuse;
19. Severe mental illness;
20. Non-compliant patients during the trial and/or follow-up period;
21. Other reasons deemed suitable for enrolment by the investigators.
