# Supplementary material for: Long-term outcomes with HLX01 (HanliKang®), a rituximab biosimilar, in previously untreated patients with diffuse large B-cell lymphoma: 5-year follow-up results of the phase 3 HLX01-NHL03 study
Source: BMC Cancer. 2024 Jan 24;24:124. doi: 10.1186/s12885-024-11876-9 (PMC10809427; doi:10.1186/s12885-024-11876-9)
Supplement: Supplementary file 9 — Supplementary Material 9 [file 12885_2024_11876_MOESM9_ESM.docx]

|  | **Survival rate (95% CI)** | **OS** | | | | **PFS** | | |
| --- | --- | --- | --- | --- | --- | --- | --- | --- |
|  |  | **H-CHOP** | | **R-CHOP** | | **H-CHOP** | | **R-CHOP** |
| **IPI score of 1 and 2** | 1-year | 93.4 (89.6-97.4) | 94.1 (90.5-97.9) | | 92.1 (87.9-96.5) | | 91.5 (87.2-96.0) | |
|  | 3-year | 88.1 (83.1-93.4) | 85.0 (79.5-90.8) | | 83.3 (77.6-89.5) | | 81.7 (75.8-88.1) | |
|  | 5-year | 81.0 (74.9-87.6) | 74.6 (67.9-81.9) | | 77.7 (71.2-84.7) | | 72.0 (65.1-79.6) | |
|  |  | **IPI 1** | **IPI 2** | | **IPI 1** | | **IPI 2** | |
| **IPI score of 1 or 2** | 1-year | 95.4 (92.2-98.8) | 92.1 (87.9-96.5) | | 94.8 (91.3-98.4) | | 88.8 (83.9-94.0) | |
|  | 3-year | 89.5 (84.8-94.5) | 83.5 (77.8-89.6) | | 84.9 (79.4-90.8) | | 80.1 (74.0-86.7) | |
|  | 5-year | 83.1 (77.3-89.4) | 72.3 (65.5-79.9) | | 79.3 (73.1-86.1) | | 70.2 (63.1-78.0) | |
|  |  | **Clinical stage I/II** | **Clinical stage III/IV** | | **Clinical stage I/II** | | **Clinical stage III/IV** | |
| **PI score of 1 and 2 stratified by clinical stage** | 1-year | 94.3 (90.7-98.0) | 93.2 (89.3-97.4) | | 93.6 (89.9-97.5) | | 89.9 (85.1-94.9) | |
|  | 3-year | 88.5 (83.6-93.6) | 84.5 (78.8-90.5) | | 85.9 (80.6-91.5) | | 79.0 (72.6-85.8) | |
|  | 5-year | 79.4 (73.1-86.2) | 76.0 (69.4-83.3) | | 77.7 (72.8-85.8) | | 70.4 (63.3-78.2) | |
